# Violence against pregnant women and associated factors in the city of Governador Valadares

**DOI:** 10.11606/s1518-8787.2020054002491

**Published:** 2020-11-27

**Authors:** Érica Cesário Defilipo, Paula Silva de Carvalho Chagas, Luiz Cláudio Ribeiro

**Affiliations:** I Universidade Federal de Juiz de Fora Instituto de Ciências da Vida Departamento de Fisioterapia Governador ValadaresMG Brasil Universidade Federal de Juiz de Fora campus Governador Valadares. Instituto de Ciências da Vida. Departamento de Fisioterapia. Governador Valadares, MG, Brasil; II Universidade Federal de Juiz de Fora Faculdade de Fisioterapia Programa de Pós-Graduação em Ciências da Reabilitação e Desempenho Físico-funcional Juiz de ForaMG Brasil Universidade Federal de Juiz de Fora. Faculdade de Fisioterapia. Programa de Pós-Graduação em Ciências da Reabilitação e Desempenho Físico-funcional. Juiz de Fora, MG, Brasil; III Universidade Federal de Juiz de Fora Departamento de Estatística Programa de Pós-Graduação em Saúde Coletiva Juiz de ForaMG Brasil Universidade Federal de Juiz de Fora. Departamento de Estatística. Programa de Pós-Graduação em Saúde Coletiva. Juiz de Fora, MG, Brasil

**Keywords:** Pregnant Women, Violence Against Women, Risk Factors, Socioeconomic Factors, Cross-Sectional Studies

## Abstract

**OBJECTIVE:**

To characterize violence against women during pregnancy and to verify its association with socioeconomic, demographic, obstetric, behavioral factors, health care and diseases during pregnancy.

**METHODS:**

Cross-sectional study carried out with puerperal women whose birth took place at the Municipal Hospital of Governador Valadares, in Minas Gerais, from May 2017 to July 2018. Data collection was performed through interviews, and complementary information was obtained by analyzing the prenatal file and medical records. For data analysis, logistic regression was used.

**RESULTS:**

The total of 771 puerperal women participated in the study. Of these, 62 (8.0%) reported having suffered physical, psychological or sexual violence during pregnancy. The pregnant women most likely to have suffered violence were alcohol dependent (OR = 4.97; 95%CI 2.30–10.75; p < 0.001), those who did not perform prenatal care (OR = 3.88; 95%CI 1.00–15.09; p = 0.050), those who used health services in an emergency during pregnancy (OR = 2.47; 95%CI 1.42–4.30; p = 0.001) and who had gestational diabetes (OR = 2.59; 95%CI 1.06–6.32; p = 0.037) and sexually transmitted diseases (OR = 3.85; 95%CI 1.41–10.50; p = 0.009).

**CONCLUSION:**

Violence against pregnant women is associated with behavioral factors and related to health care and diseases during pregnancy. It is essential to recognize factors associated by health professionals through actions to track situations of violence against women since the beginning of prenatal care, in order to enable early intervention.

## INTRODUCTION

Violence against women is recognized as a serious public health problem^1^, being defined as any act of gender-based violence that may result in physical, sexual or psychological harm or suffering to women^2^. According to the World Health Organization, about one in three women in the world has experienced violence at some point in their lives, especially physical, sexual or both^2^. This situation is aggravated when referring to women at a time of great physical and emotional fragility, such as pregnancy, as it poses a threat to the woman and the fetus, requiring greater attention from health services^1,3^.

The prevalence of violence against women during pregnancy varies between different communities, regions, and countries^4^. Most countries have a prevalence of violence during pregnancy between 2% and 13.5%, with a greater prevalence in African and Latin American countries^5^. In Brazil, the percentage of women who reported intimate partner violence ranged from about 14 to 17%^6^, with a prevalence of 8% in pregnant women^7^. Violence against women, at any stage of life, has increased considerably in recent years in the country, occurring mainly in the Southeastern, Southern and Midwestern regions^8^. In the municipality of Governador Valadares, in Minas Gerais, the rate of violence against women was 8.8% in 2017, with the highest number of victims of physical violence, followed by psychological violence^9^, with no data on the prevalence of violence against pregnant women in the municipality.

The maternal and neonatal effects of violence during pregnancy are considered preventable. For pregnant women, there is a greater risk of developing depression, insufficient weight gain during pregnancy, difficulty in carrying out prenatal care adequately and maternal death^10^. For the fetus, studies show a higher risk of low birth weight, prematurity, behavioral changes and even neonatal death^3,10,11^.

In pregnancy, there are more opportunities for screening and early intervention during routine prenatal care or hospital treatment, when necessary^10^. However, one must first identify pregnant women at risk and also those who suffer violence, since this subject is rarely reported by the victims, and the factors associated by health professionals must be recognized for possible early intervention.

This study aimed to characterize violence against women during pregnancy and to verify its association with socioeconomic, demographic, obstetric, behavioral factors, health care, and diseases during pregnancy.

## METHODS

Cross-sectional study carried out with puerperal women whose delivery took place from May 2017 to July 2018 at the Municipal Hospital of Governador Valadares, in Minas Gerais, chosen for attending the Brazilian Unified Health System and for being considered a reference for the Vale do Rio Doce region. All puerperal women participating in the study “Factors associated with prematurity and low birth weight in Governador Valadares, Minas Gerais: case-control study”^12^ were included in this study, being considered cases premature live births (gestational age below 37 weeks) and live births at term (gestational age equal to or greater than 37 weeks and less than 42 weeks) with low birth weight (less than 2,500 grams), whereas controls were those born at term with adequate weight at birth (equal to or greater than 2,500 grams), matched by sex and date of birth, with two controls selected for each case. Live births with congenital malformations, genetic syndromes, progressive diseases and injuries of the nervous system, diagnosed or suspected at birth, were excluded from the study. The study was approved by the Committee of Ethics and Research on Human Beings (CAAE: 61055716.4.0000.5147), being conducted within the required ethical standards.

Data collection was carried out through interviews with the puerperal women, still in the hospitalization period, within 24 to 48 hours after delivery, and complementary information was obtained through the analysis of the prenatal file and medical record. Data were collected by previously trained researchers. All participants read and signed both copies of the Informed Consent Form.

To have suffered physical, psychological or sexual violence during pregnancy was regarded as a dependent variable. During the interview, we asked the puerperal women: “Did you suffer physical, sexual or psychological violence during your pregnancy? In other words, did someone threaten, attack, beat, sexually abuse you, humiliate you or say something you did not like, control your behavior or make you afraid?” In the case of an affirmative answer, the type of violence suffered (physical, psychological or sexual) was also asked.

Independent variables included in the analysis were divided into five blocks: 1) socioeconomic and demographic factors; 2) obstetric factors; 3) behavioral factors; 4) factors related to the health care of pregnant women; and 5) diseases during pregnancy. The variables studied in each block were described according to the explanatory model presented in Figure 1, which contains the categorization of each variable. Detection of alcohol use was performed using the CAGE instrument (cutdown, annoyed, guilty and eye-opener)^13^, chosen because it is an instrument that is easy to apply, simple and validated for use in Brazil. Women who had an affirmative answer or more were classified as dependent on alcohol use. The categorization of the variables “number of prenatal appointments” and “first prenatal appointment” was defined based on the recommendations of the Ministry of Health, which determines the beginning of prenatal care until the sixteenth week of pregnancy and a minimum of six consultations^14^.

**Figure:**
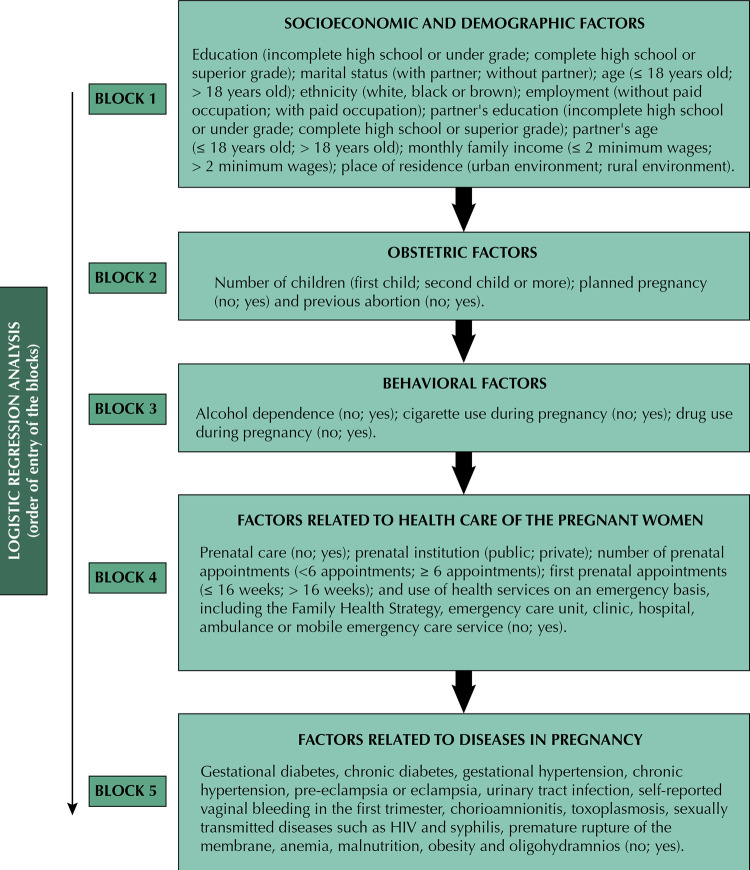
Explanatory model of independent variables divided into blocks and order of entry of factors in the logistic regression analysis.

In order to verify the associations between independent variables and violence against women during pregnancy, logistic regression was performed. The associated factors that, in the bivariate analysis, had a p-value below 0.20 were considered eligible to compose the multivariate models. Multivariate analysis of the variables in each block was performed separately, removing the variables that lost their significance. Then, the previously selected variables, which presented a p-value less than 0.05, were submitted to a new multivariate analysis, following the order of entry of the blocks: first the variables in block 1, followed by the variables in block 2, block 3, block 4, and block 5 (Figure 1).

## RESULTS

Of the 771 puerperal participants, 62 (8.0%) reported having suffered violence during pregnancy. Of these, 49 (79.0%) reported psychological violence, 11 (17.8%) physical violence, and 2 (3.2%) reported having experienced sexual violence. Among the pregnant women who were exposed to violence, there was a predominance of women with low education (incomplete high school or under grade), black or brown, without paid occupation, with a partner and with an income below two minimum wages.

In the bivariate analysis of socioeconomic and demographic factors, the following variables had p < 0.20: education (p = 0.063), age (p = 0.110) and marital status (p = 0.039). Of the obstetric factors, only the variable previous abortion (p = 0.115) had a p-value below 0.20 (Table 1).

**Table 1:** Frequency and number of pregnant women exposed or not to violence, odds ratio, 95% confidence interval and p value of socioeconomic and demographic (block 1) and obstetric (block 2) factors.

Variables	Frequency (n = 771)		Exposed to violence		Not exposed to violence		OR	95%CI	p
	n	%	n	%	n	%			
**BLOCK 1**									

Education									
Incomplete HS or under grade	393	51.0	39	62.9	354	49.9	Ref		
Complete HS or superior grade	378	49.0	23	37.1	355	50.1	0.59	0.34–1.01	0.063^b^
Age									
≤ 18 years old	99	12.8	12	19.4	87	12.3	Ref		
>18 years old	672	87.2	50	80.6	622	87.7	0.58	0.30–1.14	0.110^b^
Ethnicity									
White	111	14.4	7	11.3	104	14.7	Ref		
Black or brown	660	85.6	55	88.7	605	85.3	1.35	0.60–3.05	0.573
Marital Status									
With partner	613	79.5	43	69.4	570	80.4	Ref		
Without partner	158	20.5	19	30.6	139	19.6	1.81	1.02–3.21	0.039^b^
Employment									
Without paid occupation	503	65.2	44	71.0	459	64.7	Ref		
With paid occupation	268	34.8	18	29.0	250	35.3	0.75	0.43–1.33	0.404
Partner’s education^a^									
Incomplete HS or under grade	403	58.2	26	51.0	377	58.8	Ref		
Complete HS or superior grade	289	41.8	25	49.0	264	41.2	1.37	0.78–2.43	0.275
Partner’s age^a^									
≤ 18 years old	29	3.9	1	1.8	28	4.0	Ref		
>18 years old	720	96.1	56	98.2	664	96.0	2.36	0.32–17.68	0.718
Family income^a^									
≤ 2 minimum wages	547	74.1	45	76.3	502	73.9	Ref		
> 2 minimum wages	191	25.9	14	23.7	177	26.1	0.88	0.47–1.65	0.759
Place of residence									
Urban environment	637	82.6	55	88.7	582	82.1	Ref		
Rural environment	134	17.4	7	11.3	127	17.9	0.58	0.26–1.31	0.223

**BLOCK 2**									

Number of children									
First child	378	49.0	33	53.2	345	48.7	Ref		
Second child or more	393	51.0	29	46.8	364	51.3	0.83	0.50–1.40	0.510
Planned pregnancy									
No	429	55.6	33	53.2	396	55.9	Ref		
Yes	342	44.4	29	46.8	313	44.1	1.11	0.66–1.87	0.690
Previous abortion									
No	640	83.0	47	75.8	593	83.6	Ref		
Yes	131	17.0	15	24.2	116	16.4	1.63	0.88–3.02	0.115^b^

HS: high school; OR: odds ratio; 95%CI: 95% confidence interval; Ref: reference category

^a^ Some puerperal women did not know or did not accept to inform their partner’s data, such as education (n = 79) and age (n = 22), in addition to family income (n = 33), so these data were considered absent in the analysis.

^b^ p < 0.20.

Regarding behavioral factors, all variables analyzed were associated with violence against pregnant women: alcohol dependence (p < 0.001), cigarette use (p = 0.012) and drug use (p = 0.076). Regarding factors related to the health care of pregnant women, the variables that presented p-value below 0.20 in the bivariate analysis were: prenatal care (p = 0.001), number of appointments (p = 0.021) and emergency use of health services (p = 0.002), as shown in Table 2.

**Table 2:** Frequency and number of pregnant women exposed or not to violence, odds ratio, 95% confidence interval and p value of behavioral factors (block 3) and factors related to pregnant women health care (block 4).

Variables	Frequency (n = 771)		Exposed to violence		Not exposed to violence		OR	95%CI	Valor de p
	n	%	n	%	n	%			
**BLOCK 3**									

Alcohol dependence									
No	726	94.2	50	80.6	676	95.3	Ref		
Yes	45	5.8	12	19.4	33	4.7	4.92	2.39–10.11	< 0.001*
Cigarette use									
No	710	92.1	52	83.9	658	92.8	Ref		
Yes	61	7.9	10	16.1	51	7.2	2.48	1.19–5.17	0.012*
Drug use									
No	763	99.0	60	96.8	703	99.2	Ref		
Yes	8	1.0	2	3.2	6	0.8	3.91	0.78–19.77	0.076*

**BLOCK 4**									

Prenatal care									
No	12	1.6	4	6.5	8	1.1	Ref		
Yes	759	98.4	58	93.5	701	98.9	0.17	0.05–0.57	0.001*
Prenatal care institution									
Public	658	86.7	52	89.7	606	86.4	Ref		
Private	101	13.3	6	10.3	95	13.6	0.74	0.31–1.76	0.687
Number of prenatal appointments									
< 6 appointments	190	24.9	23	37.1	167	23.9	Ref		
≥ 6 appointments	572	75.1	39	62.9	533	76.1	0.53	0.31–0.92	0.021*
First prenatal appointments									
≤ 16 weeks	636	84.8	51	87.9	585	84.5	Ref		
> 16 weeks	114	15.2	7	12.1	107	15.5	0.75	0.33–1.70	0.572
Emergency use of health services									
No	490	63.6	28	45.2	462	65.2	Ref		
Yes	281	36.4	34	54.8	247	34.8	2.27	1.35–3.83	0.002*

OR: odds ratio; 95%CI: 95% confidence interval; Ref: reference category

* p < 0.20.

Among the diseases during pregnancy, the following variables were selected: gestational diabetes (p = 0.020), urinary tract infection (p = 0.053), vaginal bleeding in the first trimester (p = 0.003), anemia (p = 0.172), and sexually transmitted diseases (STD) (p = 0.002), as seen on Table 3.

**Table 3:** Frequency and number of pregnant women exposed or not to violence, odds ratio, 95% confidence interval and p-value of diseases during pregnancy (block 5).

Variables	Frequency (n = 771)		Exposed to violence		Not exposed to violence		OR	95%CI	p
	n	%	n	%	n	%			
Gestational diabetes									
No	732	94.9	55	88.7	677	95.5	Ref		
Yes	39	5.1	7	11.3	32	4.5	2.69	1.14–6.38	0.020*
Gestational hypertension									
No	671	87.0	53	85.5	618	87.2	Ref		
Yes	100	13.0	9	14.5	91	12.8	1.15	0.55–2.42	0.706
Chronic hypertension									
No	752	97.5	61	98.4	691	97.5	Ref		
Yes	19	2.5	1	1.6	18	2.5	0.63	0.08–4.80	1.000
Pre-eclampsia or eclampsia									
No	734	95.2	61	98.4	673	94.9	Ref		
Yes	37	4.8	1	1.6	36	5.1	0.31	0.04 - 2.27	0.352
Urinary tract infection									
No	474	61.5	31	50.0	443	62.5	Ref		
Yes	297	38.5	31	50.0	266	37.5	1.67	0.99–2.80	0.053*
Vaginal bleeding in the first trimester									
No	650	84.3	44	71.0	606	85.5	Ref		
Yes	121	15.7	18	29.0	103	14.5	2.41	1.34–4.33	0.003*
Oligohydramnios									
No	731	94.8	57	91.9	674	95.1	Ref		
Yes	40	5.2	5	8.1	35	4.9	1.69	0.64 - 4.48	0.287
Premature rupture of the membrane									
No	741	96.1	61	98.4	680	95.9	Ref		
Yes	30	3.9	1	1.6	29	4.1	0.38	0.05–2.87	0.503
Anemia									
No	555	72.0	40	64.5	515	72.6	Ref		
Yes	216	28.0	22	35.5	194	27.4	1.46	0.85–2.52	0.172*
STD (HIV or syphilis)									
No	747	96.9	56	90.3	691	97.5	Ref		
Yes	24	3.1	6	9.7	18	2.5	4.11	1.57–10.78	0.002*

STD: sexually transmitted diseases; OR: odds ratio; 95%CI: 95% confidence interval; Ref: reference category

* p < 0.20.

Variables selected by the bivariate analysis were introduced in a logistic regression model with block analysis. In the analysis of the variables in block 1, the marital status variable maintained a significant association with violence against pregnant women (p = 0.041). In block 2, the variable previous abortion lost significance. In block 3, the alcohol dependence variable showed a significant association (p < 0.001). In block 4, the variables that remained in the model were: prenatal care (p = 0.001) and emergency use of health services (p = 0.001). Finally, in block 5, the variables that maintained a significant association with violence against pregnant women were: gestational diabetes (p = 0.030), vaginal bleeding in the first trimester (p = 0.006), and STD (p = 0.014). In the logistic regression analysis, the selected variables from blocks 1 and 3 – marital status and alcohol dependence – were first introduced, with only the alcohol dependence variable being maintained in the model. Then, the variables in block 4 – prenatal care and emergency use of health services, were introduced, and both were maintained in the model. Finally, the variables in block 5 – gestational diabetes, STD, and vaginal bleeding in the first trimester, were added, and the last one was removed from the analysis because it did not present a significant association.

Table 4 presents the result of the final logistic regression model, which indicates that the variables alcohol dependence (p < 0.001), emergency use of health services (p = 0.001), gestational diabetes (p = 0.037) and STD (p = 0.009) had a significant association with violence against pregnant women. The variable prenatal care had a p-value very close to being significant, being maintained in the model for discussion (p = 0.050).

**Table 4:** Final result of logistic regression of factors associated with violence against women during pregnancy.

Blocks	Variables	OR	95%CI	p
**Block 3**	Alcohol dependence			
	No	Ref		
	Yes	4,97	2,30–10,75	< 0,001*
**Block 4**	Prenatal care			
	Yes	Ref		
	No	3,88	1,00–15,09	0,050
	Use of emergency health services			
	No	Ref		
	Yes	2,47	1,42–4,30	0,001*
**Block 5**	Gestational diabetes			
	No	Ref		
	Yes	2,59	1,06–6,32	0,037*
	Sexually Transmitted Diseases			
	No	Ref		
	Yes	3,85	1,41–10,50	0,009*

OR: odds ratio; 95%CI: 95% confidence interval; Ref: reference category

* p < 0.05.

## DISCUSSION

Violence against women during pregnancy is a major concern for global health, since not only one, but two lives are at risk^3^. In this study, 8.0% of the puerperal women interviewed reported having suffered violence during pregnancy. This fact is worrying and reinforces the need for increased attention from health services, through preventive actions and screening of violence against women since the beginning of prenatal care^1,3^.

There was no association between violence against pregnant women and socioeconomic and demographic factors. Likewise, in a Brazilian study analyzing the effects of socioeconomic level on violence against pregnant women, this factor was not associated with physical, psychological or sexual violence, having affected pregnant women at different socioeconomic levels^15^. On the other hand, another Brazilian study observed that low education increased the chance of psychological violence by 1.5 times and almost doubled the chance of physical and sexual violence^16^. Pregnant women with low education and income report more episodes of violence, which demonstrates the need to identify these women who may be at risk^15^.

Alcohol-dependent pregnant women were more likely to have been exposed to violence during pregnancy (OR = 4.97; 95%CI 2.30– 10.75; p < 0.001). It is worth mentioning that alcohol dependence in this study was assessed using a standardized instrument for screening alcohol use. The frequent use of alcohol and violence against women are interconnected, however the nature of this association is complex, since the use of alcohol can be both the cause and the consequence of violence^17^. Women can use alcohol to deal with violence, while the fact of consuming it can result in violence, in cases where the partners do not accept that the woman consumes alcohol^17^. Thus, the relationship between alcohol use and violence can be bidirectional. The consumption of alcoholic beverages is related to less union, harmony and organization in the family environment, as well as to the high levels of domestic violence^18^. This situation becomes even more worrying when we refer to the consumption of alcohol during pregnancy. The use of alcohol by pregnant women contributes to insufficient gestational weight gain, greater use of other drugs and less attendance to prenatal appointments, in addition to direct repercussions to the fetus and newborn, such as a higher risk of malformations, spontaneous abortion, prematurity, low birth weight, asphyxia, perinatal mortality and fetal alcohol syndrome^19^.

Experiences of violence during pregnancy are associated with specific behaviors or attitudes, such as inadequate prenatal care or delayed beginning of health monitoring^10,20^. In this study, pregnant women who did not undergo prenatal care were 3.8 times more likely to have suffered violence. Probably due to the small number of puerperal women who did not perform prenatal care (n = 12), the confidence interval was wide (95%CI 1.00–15.09) and the p-value was slightly higher than the reference value (p = 0.050). However, as this result showed an association very close to being significant, it deserves to be discussed in further studies, so that health professionals pay attention to this factor. Audi et al.^15^, in a study to identify factors associated with violence against pregnant women monitored in primary health care units in the city of Campinas, São Paulo, observed that the difficulty in attending prenatal appointments was associated with physical and sexual violence (OR = 2.31; 95%CI 1.18–4.51; p = 0.014). Another Brazilian study, carried out in Rio de Janeiro, found that women victims of violence delay seeking prenatal care, and those who reported having been victims of physical abuse during pregnancy were 2.2 times more likely to have inadequate prenatal care when compared to those without a history of violence^21^.

Women exposed to violence suffer constraints of various orders, such as jealousy and threats, which result in the restriction of their freedom^15^, and may also be discouraged by partners to carry out prenatal care^22^. This fact can justify the non-attendance in all scheduled consultations, as can be seen in this study, in which 6.5% of women exposed to violence did not perform prenatal care and 37.1% attended less than six consultations. Easy access to prenatal care and the development of a trusting relationship between patient and health professional are the first steps to address the problem of violence in pregnancy. Individualized interventions and home visiting programs directed at pregnant women who do not attend scheduled appointments can have promising results^9^.

Pregnant women who used health services on an emergency basis, including appointments not scheduled in the Family Health Strategy or clinics, use of ambulance or mobile emergency service, emergency care units and need for hospitalization, were more likely to having been exposed to violence (OR = 2.47; 95%CI 1.42–4.30; p = 0.001). According to the literature, this search for health services occurs due to the consequences of the high level of stress suffered by exposure to violence or due to injuries caused by possible physical aggressions, or even by sexual trauma or infections resulting from sexual violence, which can lead to complications during pregnancy^23^.

Many women, when looking for health services to take care of injuries caused by violence, are reluctant to reveal the real source of the injury, attributing it to some other cause, and most services do not collect further information^4^. New guidelines of the World Health Organization emphasize the urgent need to integrate these issues in the undergraduate curricula of all courses in the health field, as well as to train teams of the different health services to understand the relationship between violence and women health problems and to properly intervene^4^.

Intimate partner violence against women is an important contributor to women’s vulnerability to STD. In this study, women with STD were more likely to have been exposed to violence (OR = 3.85; 95%CI 1.41–10.50; p = 0.009). The mechanisms related to the increase in this vulnerability include direct infection through forced sexual intercourse, since women in violent relationships may have limited control over the moment or circumstances of sexual intercourse and little ability to negotiate condom use^4,24^.

A cohort study conducted in southern Africa found that gender inequality in relationships and intimate partner violence increase the risk of HIV infection (OR = 1.51; 95%CI 1.04–2.21; p = 0.032)^25^. The history of intimate partner violence also showed a significant association with positive syphilis test (OR = 1.61; 95%CI 1.24–2.08; p < 0.01) in Bolivian pregnant women, suggesting that this disease may be an important negative consequence of violence for the health of women and children^26^. Another study, carried out in India, aimed at describing the factors associated with the incidence of STD, found that the incidence was higher among married women and those exposed to sexual violence^27^. STD prevention policies, interventions and programs must also address this important risk factor^25^.

In addition to STD, pregnant women who had gestational diabetes were more likely to have been exposed to violence (OR = 2.59; 95%CI 1.06–6.32; p = 0.037). Gestational diabetes mellitus represents the most common metabolic problem in pregnancy^28^, and psychosocial factors and depression can contribute to its development^29^. Physical and psychological violence is associated with an increased risk of type 2 diabetes^30^, which in turn has a strong correlation with gestational diabetes, since metabolic risks may arise first in pregnancy^29^. In addition, women with a history of violence are more prone to obesity, one of the most important risk factors for gestational diabetes. Another possible biological mechanism that explains this association is that violence increases the levels of stress hormones that can trigger insulin resistance^29^. Thus, health professionals should be more alert to the identification of women at increased risk of developing gestational diabetes, which in some cases can be preventable.

The limitation of this study is the fact that no standardized instrument was used to assess violence against women, and only direct questions were asked to the puerperal women about this issue. The use of a standardized instrument could detect a greater number of puerperal women who were exposed to violence during pregnancy, but this did not affect the results. In this study, 8.0% of the puerperal women interviewed reported having suffered violence during pregnancy, a number similar to the rates found in Brazil^6^. This study did not only address intimate partner violence, as did many studies, but psychological, physical and sexual violence practiced by different people in the family environment and in the community, including the intimate partner. Another limitation of this study refers to the reverse causality relationship inherent in cross-sectional studies.

Violence against women is a public health problem with an epidemic proportion, which permeates the whole world, putting women’s health at risk, limiting their participation in society and causing great human suffering^4^. The Brazilian government, despite some initiatives such as the Maria da Penha Law, must progress in terms of legislation and action plans to combat this growing problem^7^. The debate on violence against women, especially against pregnant women, must be broaden within health services, promoting the visibility of health problems and talking about gender emancipation and the empowerment of women. Effective programs to identify victims of violence against women and intervene during pregnancy are essential, especially in primary care^15,19^.

## References

[1] 1. Baird K. Women’ s lived experience of domestic violence during pregnancy (1). Pract Midwife. 2015;18(3):27-31. 26349329

[2] 2. World Health Organization. Violence against women. Geneva: WHO; 2019 [cited 2019 Oct 20]. Available from: https://www.who.int/news-room/fact-sheets/detail/violence-against-women

[3] 3. Donovan BM, Spracklen CN, Schweizer ML, Ryckman KK, Saftlas AF. Intimate partner violence during pregnancy and the risk for adverse infant outcomes: a systematic review and meta-analysis. BJOG. 2016;123(8):1289-99. 10.1111/1471-0528.13928 26956568

[4] 4. World Health Organization, Department of Reproductive Health and Research; London School of Hygiene and Tropical Medicine; South African Medical Research Council. Global and regional estimates of violence against women: prevalence and health effects of intimate partner violence and non-partner sexual violence. Geneva: WHO; 2013 [cited 2019 Oct 20]. Available from: https://www.who.int/reproductivehealth/publications/violence/9789241564625/en/

[5] 5. Drevies KM, Kishor S, Johnson H, Stöckl H, Bacchus LJ, Garcia-Moreno C, et al. Intimate partner violence during pregnancy: analysis of prevalence data from 19 countries. Reprod Health Matters. 2010;18(36):158-70. 10.1016/S0968-8080(10)36533-5 21111360

[6] 6. Bott S, Guedes A, Ruiz-Celis AP, Mendoza JA. Intimate partner violence in the Americas: a systematic review and reanalysis of national prevalence estimates. Rev Panam Salud Publica. 2019;43:e26. 10.26633/RPSP.2019.26 PMC6425989 31093250

[7] 7. García-Moreno C, Jansen HAFM, Ellsberg M, Heise L, Watts C. WHO Multi-country Study on Women’s Health and Domestic Violence against Women: initial results on prevalence, health outcomes and women’s responses. Geneva: World Health Organization; 2005 [cited 2019 Oct 20]. Available from: https://www.who.int/reproductivehealth/publications/violence/24159358X/en/

[8] 8. Rodrigues NCP, O’Dwyer G, Andrade MKN, Flynn MB, Monteiro DLM, Lino VTS. The increase in domestic violence in Brazil from 2009-2014. Cienc Saude Coletiva. 2017;22(9):2873-80. 10.1590/1413-81232017229.09902016 28954138

[9] 9. Governo do Estado de Minas Gerais, Sistema Integrado de Defesa Social, Centro Integrado de Informações de Defesa Social. Diagnóstico de violência doméstica e familiar nas regiões integradas de segurança pública de Minas Gerais: Registros Tentados e Consumados. Belo Horizonte, MG; 2018 [cited 2019 Oct 20]. Available from: http://www.seguranca.mg.gov.br/images/2020/Maio/Diagnosticos/final_Diagnstico%20violncia%20domstica%202015%20a%202017%20-%20MG%20e%20RISPs.pdf

[10] 10. Alhusen JL, Ray E, Sharps P, Bullock L. Intimate partner violence during pregnancy: maternal and neonatal outcomes. J Women’s Health (Larchmt). 2015;24(1):100-6. 10.1089/jwh.2014.4872 PMC4361157 25265285

[11] 11. Berhanie E, Gebregziabher D, Berihu H, Gerezgiher A, Kidane G. Intimate partner violence during pregnancy and adverse birth outcomes: a case-control study. Reprod Health. 2019;16:22. 10.1186/s12978-019-0670-4 PMC6388467 30803448

[12] 12. Defilipo EC. Fatores associados à prematuridade e ao baixo peso ao nascer em Governador Valadares, Minas Gerais: estudo caso controle [tese]. Juiz de Fora, MG: Faculdade de Medicina da Universidade Federal de Juiz de Fora; 2019.

[13] 13. Mayfield D, McLeod G, Hall P. The CAGE Questionnaire: validation of a new alcoholism screening instrument. Am J Psychiatry. 1974;131(10):1121-3. 10.1176/ajp.131.10.1121 4416585

[14] 14. Ministério da Saúde (BR), Secretaria Executiva. Programa Humanização do Parto: humanização no pré-natal e nascimento. Brasília, DF; 2002 [cited 2019 Oct 20]. Available from: http://bvsms.saude.gov.br/bvs/publicacoes/parto.pdf

[15] 15. Ribeiro MRC, Silva AAM, Britto e Alves MTSS, Batista RFL, Ribeiro CCC, Schraiber LB, et al. Effects of socioeconomic status and social support on violence against pregnant women: a structural equation modeling analysis. PLoS One. 2017;12(1):e0170469. 10.1371/journal.pone.0170469 PMC5249246 28107428

[16] 16. Audi CAF, Segall-Corrêa AM, Santiago SM, Andrade MGG, Pérez-Escamilla R. Violência doméstica na gravidez : prevalência e fatores associados. Rev Saude Publica. 2008;42(5):877-85. 10.1590/S0034-89102008005000041 18695785

[17] 17. Devries KM, Child JC, Bacchus LJ, Mak J, Falder G, Graham K, et al. Intimate partner violence victimization and alcohol consumption in women: a systematic review and meta-analysis. Addiction. 2014;109(3):379-91. 10.1111/add.12393 24329907

[18] 18. Okada MM, Hoga LAK, Borges ALV, Albuquerque RS, Belli MA. Domestic violence against pregnant women. Acta Paul Enferm. 2015;28(3):270-4. 10.1590/1982-0194201500045

[19] 19. Moraes CL, Reichenheim ME. Rastreamento de uso de álcool por gestantes de serviços públicos de saúde do Rio de Janeiro. Rev Saude Pública. 2007;41(5):695-703. 10.1590/S0034-89102007000500002 17923889

[20] 20. Shrestha M, Shrestha S, Shrestha B. Domestic violence among antenatal attendees in a Kathmandu hospital and its associated factors: a cross-sectional study. BMC Pregnancy Childbirth. 2016;16(1):360. 10.1186/s12884-016-1166-7 PMC5117509 27871256

[21] 21. Moraes CL, Arana FDN, Reichenheim ME. Violência física entre parceiros íntimos na gestação como fator de risco para a má qualidade do pré-natal. Rev Saude Publica. 2010;44(4):667-76. 10.1590/S0034-89102010000400010

[22] 22. Stöckl H, Watts C, Kilonzo Mbwambo JK. Physical violence by a partner during pregnancy in Tanzania: prevalence and risk factors. Reprod Health Matters. 2010;18(36):171-80. 10.1016/S0968-8080(10)36525-6 21111361

[23] 23. Coker AL, Sanderson M, Dong B. Partner violence during pregnancy and risk of adverse pregnancy outcomes. Paediatr Perinat Epidemiol. 2004;18(4):260-9. 10.1111/j.1365-3016.2004.00569.x 15255879

[24] 24. Chambliss LR. Intimate partner violence and its implication for pregnancy. Clin Obstet Gynecol. 2008;51(2):385-97. 10.1097/GRF.0b013e31816f29ce 18463468

[25] 25. Jewkes RK, Dunkle K, Nduna M, Shai N. Intimate partner violence, relationship power inequity, and incidence of HIV infection in young women in South Africa: a cohort study. Lancet. 2010;376(9734):41-8. 10.1016/S0140-6736(10)60548-X 20557928

[26] 26. Díáz-Olavarrieta C, Wilson KS, García SG, Revollo R, Richmond K, Paz F, et al. The co-occurrence of intimate partner violence and syphilis among pregnant women in Bolivia. J Women’s Health (Larchmt). 2009;18(12):2077-86. 10.1089/jwh.2008.1258 20044873

[27] 27. Weiss HA, Patel V, West B, Peeling RW, Kirkwood BR, Mabey D. Spousal sexual violence and poverty are risk factors for sexually transmitted infections in women: a longitudinal study of women in Goa, India. Sex Transm Infect. 2008;84(2):133-9. 10.1136/sti.2007.026039 17942576

[28] 28. Wilson BL, Dyer JM, Latendresse G, Wong B, Baksh L. Exploring the psychosocial predictors of gestational diabetes and birth weight. J Obstet Gynecol Neonatal Nurs. 2015;44(6):760-71. 10.1111/1552-6909.12754 26402777

[29] 29. Mason SM, Tobias DK, Clark CJ, Zhang C, Hu FB, Rich-Edwards JW. Abuse in Childhood or Adolescence and Gestational Diabetes: a retrospective cohort study. Am J Prev Med. 2016;50(4):436-44. 10.1016/j.amepre.2015.08.033 PMC4801767 26547539

[30] 30. Mason SM, Wright RJ, Hibert EN, Spiegelman D, Jun HJ, Hu FB, et al. Intimate partner violence and incidence of type 2 diabetes in women. Diabetes Care. 2013;36(5):1159-65. 10.2337/dc12-1082 PMC3631851 23248189

