# Supplementary material for: Violence against pregnant women and associated factors in the city of Governador Valadares
Source: Rev Saude Publica. 2020 Nov 27;54:135. doi: 10.11606/s1518-8787.2020054002491 (PMC7702383; doi:10.11606/s1518-8787.2020054002491)
Supplement: Translation

# Translation: Violência contra a gestante e fatores associados no município de Governador Valadares

**Authors:** Érica Cesário Defilipo, Paula Silva de Carvalho Chagas, Luiz Cláudio Ribeiro

**Affiliations:** I Brasil Universidade Federal de Juiz de Fora campus Governador Valadares. Instituto de Ciências da Vida. Departamento de Fisioterapia. Governador Valadares, MG, Brasil; II Brasil Universidade Federal de Juiz de Fora. Faculdade de Fisioterapia. Programa de Pós-Graduação em Ciências da Reabilitação e Desempenho Físico-funcional. Juiz de Fora, MG, Brasil; III Brasil Universidade Federal de Juiz de Fora. Departamento de Estatística. Programa de Pós-Graduação em Saúde Coletiva. Juiz de Fora, MG, Brasil

**Keywords:** Gestantes, Violência contra a Mulher, Fatores de Risco, Fatores Socioeconômicos, Estudos Transversais

## Abstract

**OBJETIVO:**

Caracterizar a violência contra a mulher durante a gestação e verificar a associação com os fatores socioeconômicos, demográficos, obstétricos, comportamentais, de assistência à saúde e doenças na gestação.

**MÉTODOS:**

Estudo transversal realizado com puérperas cujo parto ocorreu no Hospital Municipal de Governador Valadares, em Minas Gerais, no período de maio de 2017 a julho de 2018. A coleta de dados foi realizada por meio de entrevista, e informações complementares foram obtidas pela análise do cartão de pré-natal e prontuário. Para a análise dos dados, foi utilizada regressão logística.

**RESULTADOS:**

Participaram do estudo 771 puérperas. Dessas, 62 (8,0%) relataram ter sofrido violência física, psicológica ou sexual durante a gestação. As gestantes com maior chance de terem sofrido violência foram as dependentes de álcool (RC = 4,97; IC95% 2,30–10,75; p < 0,001), as que não realizaram o pré-natal (RC = 3,88; IC95% 1,00–15,09; p = 0,050), as que usaram os serviços de saúde de forma emergencial durante a gravidez (RC = 2,47; IC95% 1,42–4,30; p = 0,001) e que apresentaram diabetes gestacional (RC = 2,59; IC95% 1,06–6,32; p = 0,037) e doenças sexualmente transmissíveis (RC = 3,85; IC95% 1,41–10,50; p = 0,009).

**CONCLUSÃO:**

A violência contra a gestante está associada a fatores comportamentais e relacionados à assistência à saúde e doenças na gestação. É imprescindível o reconhecimento dos fatores associados pelos profissionais de saúde, por meio de ações de rastreamento de situações de violência contra a mulher desde o início do pré-natal, a fim de possibilitar intervenção precoce.

## INTRODUÇÃO

A violência contra a mulher é reconhecida como grave problema de saúde pública^1^, sendo definida como qualquer ato de violência de gênero que possa resultar em danos físicos, sexuais ou psicológicos ou sofrimento às mulheres^2^. De acordo com a Organização Mundial de Saúde, cerca de uma em cada três mulheres no mundo já sofreram violência em algum momento da vida, em especial física, sexual ou ambas^2^. Tal situação agrava-se quando se refere à mulher em um momento de grande fragilidade física e emocional como a gestação, pois apresenta uma ameaça para a mulher e para o feto, exigindo maior atenção dos serviços de saúde^1,3^.

A prevalência de violência contra a mulher durante a gestação varia entre diferentes comunidades, regiões e países^4^. A maioria dos países apresenta prevalências de violência na gestação entre 2% e 13,5%, com maior predomínio nos países da África e da América Latina^5^. No Brasil, a proporção de mulheres que relataram violência por parceiro íntimo variou de cerca de 14 a 17%^6^, com prevalência de 8% nas gestantes^7^. A violência contra a mulher, em qualquer fase da vida, aumentou consideravelmente nos últimos anos no país, ocorrendo principalmente no Sudeste, Sul e Centro-Oeste^8^. No município de Governador Valadares, em Minas Gerais, a taxa de violência contra a mulher foi de 8,8% no ano de 2017, com maior quantitativo de vítimas de violência física, seguida da violência psicológica^9^, não havendo dados sobre a prevalência de violência contra a gestante no município.

Os efeitos maternos e neonatais da violência na gestação são considerados preveníveis. Para a gestante há maior risco de desenvolver depressão, ganho de peso insuficiente na gestação, dificuldade de realizar pré-natal de forma adequada e morte materna^10^. Para o feto, estudos demonstram maior risco de baixo peso ao nascer, prematuridade, alterações comportamentais e até morte neonatal^3,10,11^.

Na gravidez existem mais oportunidades para a triagem e intervenção precoce durante o pré-natal de rotina ou tratamento hospitalar, quando necessário^10^. No entanto, é preciso primeiramente identificar as gestantes em risco e também aquelas que sofrem violência, visto que esse assunto raramente é reportado pelas vítimas, sendo necessário o reconhecimento dos fatores associados pelos profissionais de saúde para possível intervenção precoce.

Este estudo objetivou caracterizar a violência contra a mulher durante a gestação e verificar a associação com os fatores socioeconômicos, demográficos, obstétricos, comportamentais, de assistência à saúde e doenças na gestação.

## MÉTODOS

Estudo transversal, realizado com puérperas cujo parto ocorreu no período de maio de 2017 a julho de 2018 no Hospital Municipal de Governador Valadares, em Minas Gerais, escolhido por atender ao Sistema Único de Saúde e por ser considerado referência para a região do Vale do Rio Doce. Foram incluídas no estudo todas as puérperas participantes da pesquisa “Fatores associados à prematuridade e ao baixo peso ao nascer em Governador Valadares, Minas Gerais: estudo caso-controle”^12^, no qual foram considerados casos os nascidos vivos prematuros (idade gestacional inferior a 37 semanas) e também os nascidos vivos a termo (idade gestacional igual ou superior a 37 semanas e inferior a 42 semanas) com baixo peso ao nascer (inferior a 2.500 gramas), enquanto os controles foram os nascidos vivos a termo com peso adequado ao nascer (igual ou superior a 2.500 gramas), pareados pelo sexo e data de nascimento, sendo selecionados dois controles para cada caso. Foram excluídos do estudo os nascidos vivos com malformações congênitas, síndromes genéticas, doenças progressivas e lesões do sistema nervoso, diagnosticadas ou suspeitas ao nascimento. O estudo foi aprovado pelo Comitê de Ética e Pesquisa em Seres Humanos (CAAE: 61055716.4.0000.5147), sendo conduzido dentro dos padrões éticos exigidos.

A coleta de dados foi realizada por meio de entrevista com as puérperas, ainda no período de internação hospitalar, em um prazo de 24 a 48 horas após o parto, e informações complementares foram obtidas por meio da análise do cartão de pré-natal e do prontuário da puérpera. Os dados foram coletados por pesquisadores previamente treinados. Todas as participantes assinaram o termo de consentimento livre e esclarecido

Foi considerada como variável dependente ter sofrido violência física, psicológica ou sexual na gravidez. Durante a entrevista, era perguntado à puérpera: “Você sofreu violência física, sexual ou psicológica durante sua gestação? Ou seja, alguém te ameaçou, agrediu, bateu, abusou sexualmente de você, te humilhou ou falou algo que você não gostou, controlou seu comportamento ou te deixou com medo?” No caso de resposta afirmativa, também foi questionado qual o tipo de violência sofrida (física, psicológica ou sexual).

As variáveis independentes incluídas na análise foram divididas em cinco blocos: 1) fatores socioeconômicos e demográficos; 2) fatores obstétricos; 3) fatores comportamentais; 4) fatores relacionados à assistência à saúde da gestante; e 5) doenças na gestação. As variáveis estudadas em cada bloco foram descritas de acordo com o modelo explicativo apresentado na Figura 1, na qual consta a forma de categorização de cada variável. A detecção do uso de álcool foi realizada por meio do instrumento CAGE (*cutdown, annoyed, guilty e eye-opener*)^13^, escolhido por ser um instrumento de fácil aplicação, simples e com validade para uso no Brasil. Foram classificadas como dependentes do uso de álcool as mulheres que apresentaram uma resposta afirmativa ou mais. A categorização das variáveis “número de consultas de pré-natal” e “primeira consulta de pré-natal” foi definida com base no preconizado pelo Ministério da Saúde, que determina o início da assistência pré-natal até a décima sexta semana de gestação e mínimo de seis consultas^14^.

**Figura:** Modelo explicativo das variáveis independentes divididas em blocos e ordem de entrada dos fatores na análise de regressão logística.

Para verificar as associações das variáveis independentes com a violência contra a mulher durante a gestação, foi realizada regressão logística. Os fatores associados que, na análise bivariada, apresentaram p-valor inferior a 0,20 foram considerados elegíveis para compor os modelos multivariados. Foi realizada análise multivariada das variáveis de cada bloco separadamente, retirando as variáveis que perderam a significância. Em seguida, as variáveis anteriormente selecionadas, que apresentaram p-valor inferior a 0,05, foram submetidas a nova análise multivariada, seguindo a ordem de entrada dos blocos: primeiro as variáveis do bloco 1, seguidas das variáveis do bloco 2, bloco 3, bloco 4 e bloco 5 (Figura 1).

## RESULTADOS

Das 771 puérperas participantes, 62 (8,0%) relataram ter sofrido violência durante a gestação. Dessas, 49 (79,0%) relataram violência psicológica, 11 (17,8%) violência física e 2 (3,2%) relataram ter sofrido violência sexual. Entre as gestantes que foram expostas à violência, houve predomínio de mulheres com baixa escolaridade (ensino médio incompleto ou menos), negras ou pardas, sem ocupação remunerada, com companheiro e com renda inferior a dois salários mínimos.

Na análise bivariada dos fatores socioeconômicos e demográficos, apresentaram p < 0,20 as seguintes variáveis: escolaridade (p = 0,063), idade (p = 0,110) e situação conjugal (p = 0,039). Dos fatores obstétricos, apenas a variável ter sofrido aborto prévio (p = 0,115) apresentou p-valor inferior a 0,20 (Tabela 1).

**Tabela 1:** Frequência e número de gestantes expostas ou não à violência, razão de chances, intervalo de confiança de 95% e valor de p dos fatores socioeconômicos e demográficos (bloco1) e obstétricos (bloco 2).

Variáveis	Frequência (n = 771)		Expostas à violência		Não expostas à violência		RC	IC95%	p
	n	%	n	%	n	%			
**BLOCO 1**									

**Escolaridade**									
EM incompleto ou menos	393	51,0	39	62,9	354	49,9	Ref		
EM completo ou mais	378	49,0	23	37,1	355	50,1	0,59	0,34–1,01	0,063^b^
**Idade**									
≤ 18 anos	99	12,8	12	19,4	87	12,3	Ref		
> 18 anos	672	87,2	50	80,6	622	87,7	0,58	0,30–1,14	0,110^b^
**Raça**									
Branca	111	14,4	7	11,3	104	14,7	Ref		
Negra ou parda	660	85,6	55	88,7	605	85,3	1,35	0,60–3,05	0,573
**Situação conjugal**									
Com companheiro	613	79,5	43	69,4	570	80,4	Ref		
Sem companheiro	158	20,5	19	30,6	139	19,6	1,81	1,02–3,21	0,039^b^
**Ocupação**									
Sem ocupação remunerada	503	65,2	44	71,0	459	64,7	Ref		
Com ocupação remunerada	268	34,8	18	29,0	250	35,3	0,75	0,43–1,33	0,404
**Escolaridade do companheiro**^a^									
EM incompleto ou menos	403	58,2	26	51,0	377	58,8	Ref		
EM completo ou mais	289	41,8	25	49,0	264	41,2	1,37	0,78–2,43	0,275
**Idade do companheiro**^a^									
≤ 18 anos	29	3,9	1	1,8	28	4,0	Ref		
> 18 anos	720	96,1	56	98,2	664	96,0	2,36	0,32–17,68	0,718
**Renda familiar**^a^									
≤ 2 salários mínimos	547	74,1	45	76,3	502	73,9	Ref		
> 2 salários mínimos	191	25,9	14	23,7	177	26,1	0,88	0,47–1,65	0,759
**Local de residência**									
Urbano	637	82,6	55	88,7	582	82,1	Ref		
Rural	134	17,4	7	11,3	127	17,9	0,58	0,26–1,31	0,223

**BLOCO 2**									

**Número de filhos**									
Primeiro filho	378	49,0	33	53,2	345	48,7	Ref		
Segundo filho ou mais	393	51,0	29	46,8	364	51,3	0,83	0,50–1,40	0,510
**Gravidez planejada**									
Não	429	55,6	33	53,2	396	55.9	Ref		
Sim	342	44,4	29	46,8	313	44,1	1,11	0,66–1,87	0,690
**Aborto prévio**									
Não	640	83,0	47	75,8	593	83,6	Ref		
Sim	131	17,0	15	24,2	116	16,4	1,63	0,88–3,02	0,115^b^

EM: ensino médio; RC: razão de chances; IC95%: intervalo de confiança de 95%; Ref: categoria de referência

^a^ Algumas puérperas não souberam ou não aceitaram informar os dados do companheiro, como escolaridade (n = 79) e idade (n = 22), além da renda familiar (n = 33), portanto esses dados foram considerados ausentes na análise.

^b^ p < 0,20.

Com relação aos fatores comportamentais, todas as variáveis analisadas apresentaram associação com a violência contra a gestante: dependência de álcool (p < 0,001), uso de cigarro (p = 0,012) e uso de drogas (p = 0,076). Já no que diz respeito aos fatores relacionados à assistência à saúde da gestante, apresentaram p-valor inferior a 0,20, na análise bivariada, as variáveis: realização de pré-natal (p = 0,001), número de consultas (p = 0,021) e uso emergencial dos serviços de saúde (p = 0,002), como se observa na Tabela 2.

**Tabela 2:** Frequência e número de gestantes expostas ou não à violência, razão de chances, intervalo de confiança 95% e valor de p dos fatores comportamentais (bloco 3) e fatores relacionados à assistência à saúde da gestante (bloco 4).

Variáveis	Frequência (n = 771)		Expostas à violência		Não expostas à violência		RC	IC95%	p
	n	%	n	%	n	%			
**BLOCO 3**									

**Dependência de álcool**									
Não	726	94,2	50	80,6	676	95,3	Ref		
Sim	45	5,8	12	19,4	33	4,7	4,92	2,39–10,11	< 0,001*
**Uso de cigarro**									
Não	710	92,1	52	83,9	658	92,8	Ref		
Sim	61	7,9	10	16,1	51	7,2	2,48	1,19–5,17	0,012*
**Uso de drogas**									
Não	763	99,0	60	96,8	703	99,2	Ref		
Sim	8	1,0	2	3,2	6	0,8	3,91	0,78–19,77	0,076*

**BLOCO 4**									

**Realização do pré-natal**									
Não	12	1,6	4	6,5	8	1,1	Ref		
Sim	759	98,4	58	93,5	701	98,9	0,17	0,05–0,57	0,001*
**Local do pré-natal**									
Público	658	86,7	52	89,7	606	86,4	Ref		
Privado	101	13,3	6	10,3	95	13,6	0,74	0,31–1,76	0,687
**Número de consultas de pré-natal**									
< 6 consultas	190	24,9	23	37,1	167	23,9	Ref		
≥ 6 consultas	572	75,1	39	62,9	533	76,1	0,53	0,31–0,92	0,021*
**Primeira consulta de pré-natal**									
≤ 16 semanas	636	84,8	51	87,9	585	84,5	Ref		
> 16 semanas	114	15,2	7	12,1	107	15,5	0,75	0,33–1,70	0,572
**Uso emergencial dos serviços de saúde**									
Não	490	63,6	28	45,2	462	65,2	Ref		
Sim	281	36,4	34	54,8	247	34,8	2,27	1,35–3,83	0,002*

RC: razão de chances; IC95%: intervalo de confiança de 95%; Ref: categoria de referência

* p < 0,20.

Dentre as doenças na gestação, foram selecionadas as variáveis: diabetes gestacional (p = 0,020), infecção do trato urinário (p = 0,053), sangramento vaginal no primeiro trimestre (p = 0,003), anemia (p = 0,172) e doenças sexualmente transmissíveis (DST) (p = 0,002), conforme a Tabela 3.

**Tabela 3:** Frequência e número de gestantes expostas ou não à violência, razão de chances, intervalo de confiança 95% e valor de p das doenças na gestação (bloco 5).

Variáveis	Frequência (n = 771)		Expostas à violência		Não expostas à violência		RC	IC95%	p
	n	%	n	%	n	%			
**Diabetes gestacional**									
Não	732	94,9	55	88,7	677	95,5	Ref		
Sim	39	5,1	7	11,3	32	4,5	2,69	1,14–6,38	0,020*
**Hipertensão gestacional**									
Não	671	87,0	53	85,5	618	87,2	Ref		
Sim	100	13,0	9	14,5	91	12,8	1,15	0,55–2,42	0,706
**Hipertensão crônica**									
Não	752	97,5	61	98,4	691	97,5	Ref		
Sim	19	2,5	1	1,6	18	2,5	0,63	0,08–4,80	1,000
**Pré-eclâmpsia ou eclâmpsia**									
Não	734	95,2	61	98,4	673	94,9	Ref		
Sim	37	4,8	1	1,6	36	5,1	0,31	0,04 - 2,27	0,352
**Infecção do trato urinário**									
Não	474	61,5	31	50,0	443	62,5	Ref		
Sim	297	38,5	31	50,0	266	37,5	1,67	0,99–2,80	0,053*
**Sangramento vaginal no primeiro trimestre**									
Não	650	84,3	44	71,0	606	85,5	Ref		
Sim	121	15,7	18	29,0	103	14,5	2,41	1,34–4,33	0,003*
**Oligodrâmnio**									
Não	731	94,8	57	91,9	674	95,1	Ref		
Sim	40	5,2	5	8,1	35	4,9	1,69	0,64 - 4,48	0,287
**Ruptura prematura da membrana**									
Não	741	96,1	61	98,4	680	95,9	Ref		
Sim	30	3,9	1	1,6	29	4,1	0,38	0,05–2,87	0,503
**Anemia**									
Não	555	72,0	40	64,5	515	72,6	Ref		
Sim	216	28,0	22	35,5	194	27,4	1,46	0,85–2,52	0,172*
**DST (HIV ou sífilis)**									
Não	747	96,9	56	90,3	691	97,5	Ref		
Sim	24	3,1	6	9,7	18	2,5	4,11	1,57–10,78	0,002*

DST: doenças sexualmente transmissíveis; RC: razão de chances; IC95%: intervalo de confiança de 95%; Ref: categoria de referência

* p < 0,20.

As variáveis selecionadas pela análise bivariada foram introduzidas em um modelo de regressão logística com análise por blocos. Na análise das variáveis do bloco 1, a variável situação conjugal manteve associação significativa com a violência contra a gestante (p = 0,041). No bloco 2, a variável ter sofrido aborto prévio perdeu significância. Já no bloco 3, a variável dependência de álcool apresentou associação significativa (p < 0,001). No bloco 4, as variáveis que permaneceram no modelo foram: realização do pré-natal (p = 0,001) e uso emergencial dos serviços de saúde (p = 0,001). Por fim, no bloco 5, as variáveis que mantiveram associação significativa com a violência contra a gestante foram: diabetes gestacional (p = 0,030), sangramento vaginal no primeiro trimestre (p = 0,006) e DST (p = 0,014). Na análise de regressão logística foram introduzidas, primeiramente, as variáveis selecionadas dos blocos 1 e 3, situação conjugal e dependência de álcool, sendo mantida no modelo apenas a variável dependência de álcool. Em seguida, foram introduzidas as variáveis do bloco 4, realização do pré-natal e uso emergencial dos serviços de saúde, e ambas foram mantidas no modelo. Por fim, foram acrescentadas as variáveis do bloco 5, diabetes gestacional, DSTs e sangramento vaginal no primeiro trimestre, e a última foi retirada da análise por não apresentar associação significativa.

A Tabela 4 apresenta o resultado do modelo final da regressão logística, que indica que as variáveis dependência de álcool (p < 0,001), uso emergencial dos serviços de saúde (p = 0,001), diabetes gestacional (p = 0,037) e DST (p = 0,009) apresentaram associação significativa com a violência contra a gestante. A variável realização de pré-natal apresentou p-valor bem próximo de ser significativo, sendo mantida no modelo para discussão (p = 0,050).

**Tabela 4:** Resultado final da regressão logística dos fatores associados à violência contra a mulher na gestação.

Blocos	Variáveis	RC	IC95%	p
**Bloco 3**	**Dependência de álcool**			
	Não	Ref		
	Sim	4,97	2,30–10,75	< 0,001*
**Bloco 4**	**Realização do pré-natal**			
	Sim	Ref		
	Não	3,88	1,00–15,09	0,050
	**Uso de serviços de saúde emergencial**			
	Não	Ref		
	Sim	2,47	1,42–4,30	0,001*
**Bloco 5**	**Diabetes gestacional**			
	Não	Ref		
	Sim	2,59	1,06–6,32	0,037*
	**Doenças sexualmente transmissíveis**			
	Não	Ref		
	Sim	3,85	1,41–10,50	0,009*

RC: razão de chances; IC95%: intervalo de confiança de 95%; Ref: categoria de referência

* p < 0,05.

## DISCUSSÃO

A violência contra a mulher durante a gestação é motivo de grande preocupação de saúde global, visto que não apenas uma, mas duas vidas estão em risco^3^. Neste estudo, 8,0% das puérperas entrevistadas relataram ter sofrido violência durante a gestação. Tal fato é preocupante e reforça a necessidade de atenção redobrada dos serviços de saúde por meio de ações preventivas e de rastreamento de situações de violência contra a mulher desde o início do pré-natal^1,3^.

Não foi observada associação entre violência contra a gestante e fatores socioeconômicos e demográficos. Da mesma forma, em um estudo brasileiro que analisou os efeitos do nível socioeconômico sobre a violência contra a mulher grávida, esse fator não esteve associado à violência física, psicológica ou sexual, tendo afetado gestantes de diferentes níveis socioeconômicos^15^. Por outro lado, outro estudo brasileiro observou que a baixa escolaridade aumentou em 1,5 vez a chance de violência psicológica e quase dobrou a chance de violência física e sexual^16^. As gestantes com baixa escolaridade e renda relatam mais episódios de violência, o que demonstra a necessidade de identificação dessas mulheres que podem estar em risco^15^.

As gestantes dependentes de álcool apresentaram maior chance de terem sido expostas à violência durante a gestação (RC = 4,97; IC95% 2,30–10,75; p < 0,001). Vale ressaltar que a dependência de álcool neste estudo foi avaliada por meio de um instrumento padronizado para rastreio do uso de álcool. O uso frequente de álcool e a violência contra a mulher estão interligados, porém a natureza dessa associação é complexa, visto que o uso de álcool pode ser tanto a causa quanto a consequência da violência^17^. As mulheres podem utilizar a bebida para lidar com a violência, ao mesmo tempo que o fato de consumi-la pode resultar em violência, nos casos em que os parceiros não aceitam que a mulher consuma bebida alcóolica^17^. Dessa forma, a relação do uso de álcool e violência pode ser bidirecional. O consumo de bebida alcoólica está relacionado à menor união, harmonia e organização no ambiente familiar, assim como aos altos níveis de violência doméstica^18^. Tal situação torna-se ainda mais preocupante quando nos referimos ao consumo de bebida alcóolica durante a gestação. O uso de álcool pela gestante contribui para o ganho de peso gestacional insuficiente, maior utilização de outras drogas e menor comparecimento às consultas de pré-natal, além das repercussões diretas ao feto e recém-nascido, como maior risco de malformações, aborto espontâneo, prematuridade, baixo peso ao nascer, asfixia, mortalidade perinatal e síndrome do alcoolismo fetal^19^.

Experiências de violência durante a gestação estão associadas a comportamentos ou atitudes específicas, como o cuidado pré-natal inadequado ou início do acompanhamento tardio^10,20^. No presente estudo, as gestantes que não realizaram o pré-natal apresentaram 3,8 vezes mais chances de terem sofrido violência. Provavelmente devido ao pequeno número de puérperas que não realizaram o pré-natal (n = 12), o intervalo de confiança foi amplo (IC95% 1,00–15,09) e o p-valor foi ligeiramente superior ao valor de referência (p = 0,050). No entanto, como esse resultado apresentou associação bem próxima de ser significativa, merece ser discutido em estudos futuros, a fim de que os profissionais de saúde se atentem para esse fator. Audi et al.^15^, em estudo para identificar os fatores associados à violência contra gestantes acompanhadas em unidades de atenção primária à saúde no município de Campinas, São Paulo, observaram que a dificuldade em comparecer às consultas de pré-natal foi associada à violência física e sexual (RC = 2,31; IC95% 1,18–4,51; p = 0,014). Outro estudo brasileiro, realizado no Rio de Janeiro, identificou que mulheres vítimas de violência buscam pela assistência pré-natal tardiamente, e as que relataram ter sido vítimas de abuso físico durante a gestação possuíam 2,2 vezes mais chance de apresentar uma assistência pré-natal inadequada do que aquelas sem história de violência^21^.

Mulheres expostas à violência sofrem constrangimentos de várias ordens, como ciúmes e ameaças, que resultam em restrição de sua liberdade^15^, podendo também ser desencorajadas pelos parceiros a realizarem o acompanhamento pré-natal^22^. Tal fato pode justificar a não realização ou não comparecimento em todas as consultas agendadas, conforme pode ser observado neste estudo, no qual 6,5% das mulheres expostas à violência não realizaram o pré-natal e 37,1% compareceram a menos de seis consultas. A facilidade de acesso à assistência pré-natal e o desenvolvimento de uma relação de confiança entre paciente e profissional de saúde são os primeiros passos para abordar o problema da violência na gravidez. Intervenções individualizadas e programas de visitas domiciliares direcionadas às gestantes que não comparecem às consultas agendadas podem ter resultados promissores^9^.

As gestantes que utilizaram os serviços de saúde de forma emergencial, incluindo consultas não agendadas na Estratégia Saúde da Família ou clínicas, uso de ambulância ou serviço de atendimento móvel de urgência, unidades de pronto atendimento e necessidade de internação em hospital, apresentaram maior chance de terem sido expostas à violência (RC = 2,47; IC95% 1,42–4,30; p = 0,001). De acordo com a literatura, essa procura por serviços de saúde ocorre devido às consequências do elevado nível de estresse sofrido pela exposição à violência ou devido às lesões causadas pelas possíveis agressões físicas, ou ainda pelo trauma sexual ou infecções resultantes da violência sexual, o que pode acarretar complicações na gestação^23^.

Muitas mulheres, quando procuram o serviço de saúde para cuidar de lesões causadas por violência, relutam em revelar a fonte real da lesão, atribuindo-a a alguma outra causa, e a maioria dos serviços não coletam maiores informações^4^. As novas diretrizes da Organização Mundial da Saúde enfatizam a urgente necessidade de integrar essas questões nos currículos de graduação de todos os cursos da área da saúde, bem como treinar as equipes dos diversos serviços de saúde para entender a relação entre violência e problemas de saúde das mulheres e para intervir de forma adequada^4^.

A violência contra a mulher por parceiro íntimo é um importante contribuinte para a vulnerabilidade das mulheres às DST. Neste estudo, as mulheres com DST apresentaram maior chance de terem sido expostas à violência (RC = 3,85; IC95% 1,41–10,50; p = 0,009). Os mecanismos relacionados ao aumento dessa vulnerabilidade incluem a infecção direta por meio de relações sexuais forçadas, visto que mulheres em relacionamentos violentos podem ter controle limitado sobre o momento ou as circunstâncias da relação sexual e pouca capacidade de negociar o uso de preservativo^4,24^.

Um estudo de coorte realizado no sul da África encontrou que a desigualdade de gênero nos relacionamentos e a violência por parceiro íntimo aumentam o risco de infecção por HIV (RC = 1,51; IC95% 1,04–2,21; p = 0,032)^25^. A história de violência por parceiro íntimo também apresentou associação significativa com o teste de sífilis positivo (RC = 1,61; IC95% 1,24–2,08; p < 0,01) em gestantes bolivianas, sugerindo que essa doença pode ser uma importante consequência negativa da violência para a saúde da mulher e da criança^26^. Outro estudo realizado na Índia, com o objetivo de descrever os fatores associados à incidência de DST, verificou que a incidência foi mais alta entre as mulheres casadas e expostas à violência sexual^27^. As políticas, intervenções e programas de prevenção das DST devem abordar também esse importante fator de risco^25^.

Além das DST, as gestantes que apresentaram diabetes gestacional tiveram maior chance de terem sido expostas à violência (RC = 2,59; IC95% 1,06–6,32; p = 0,037). A diabetes mellitus gestacional representa o problema metabólico mais comum na gestação^28^, sendo que os fatores psicossociais e depressão podem contribuir para o seu desenvolvimento^29^. A violência física e psicológica está associada ao maior risco de diabetes tipo 2^30^, que por sua vez tem forte correlação com a diabetes gestacional, visto que os riscos metabólicos podem surgir primeiro na gravidez^29^. Além disso, mulheres com histórias de violência estão mais propensas à obesidade, um dos fatores de risco mais importante para a diabetes gestacional. Outro possível mecanismo biológico que explica essa associação é que a violência aumenta os níveis de hormônios do estresse que podem desencadear resistência à insulina^29^. Assim, os profissionais de saúde devem estar mais alertas para a identificação das mulheres com risco aumentado para desenvolver diabetes gestacional, que em alguns casos pode ser prevenível.

Este estudo apresenta como limitação o fato de não ter sido utilizado nenhum instrumento padronizado para avaliar a violência contra mulher, sendo realizadas apenas perguntas diretas às puérperas sobre essa questão. O uso de um instrumento padronizado poderia detectar um maior número de puérperas que foram expostas à violência durante a gestação, mas isso não prejudicou os resultados. Neste estudo, 8,0% das puérperas entrevistadas relataram ter sofrido violência durante a gestação, número semelhante às taxas encontradas no Brasil^6^. Este estudo não abordou apenas a violência por parceiro íntimo, assim como muitos estudos, mas a violência psicológica, física e sexual praticada por diferentes pessoas no ambiente familiar e na comunidade, incluindo também o parceiro íntimo. Outra limitação deste estudo refere-se à relação de causalidade reversa inerente aos estudos transversais.

A violência contra a mulher é um problema de saúde pública com proporção epidêmica, que permeia todo o mundo, colocando a saúde das mulheres em risco, limitando sua participação na sociedade e causando grande sofrimento humano^4^. O governo brasileiro, apesar de algumas iniciativas como a Lei Maria da Penha, deve progredir em termos de legislação e planos de ação para combater esse problema crescente^7^. É preciso ampliar o debate acerca da violência contra a mulher, em especial as gestantes, dentro dos serviços de saúde, promovendo a visibilidade dos agravos e dialogando sobre a emancipação de gênero e o empoderamento das mulheres. Programas efetivos para identificar as vítimas de violência contra a mulher e intervir ainda durante a gestação são fundamentais, especialmente na atenção primária^15,19^.
